# Emotional distress and its predictors in burn patients

**Published:** 2019-08

**Authors:** Homayoun Sadeghi-Bazargani, Zahra Zare, Fatemeh Ranjbar

**Affiliations:** ^*a*^Associate Professor of Epidemiology MD PhD, Road Traffic Injury Research Center, Tabriz University of Medical Sciences, Tabriz,Iran.; ^*b*^Epidemiologist, Road Traffic Injury Research Center, Tabriz University of Medical Sciences, Tabriz, Iran.; ^*c*^Professor of Psychiatry MD, Research center of Psychiatry and Behavioral sciences, Tabriz University of Medical Sciences, Tabriz, Iran.

## Abstract

**Background:**

Burn injuries are most certainly stressful events, particularly when permanent disfigurement is a result. Survivors of burn events are even more susceptible to psychological disorders compared to other groups. Purposes Present study was conducted to assess predictors of emotional distress and possible psychiatric morbidity in burn patients.

**Methods:**

This cross-sectional study was carried out on a total of 255 burn patients. In order to assess emotional distress and irrational beliefs, General Health Questionnaire (GHQ) and Scale for Irrational Thoughts after Burning (SITB) was used. To identify predictors of emotional distress, both bivariate and multivariate analysis method were conducted. In multivariate linear regression, forward strategy was used for building the model. Preliminary variable selection for model design was based on a P<0.2 and final decision for keeping the variables in the model was based on a P< 0.05.

**Results:**

The results of bivariate analysis showed that gender, occupation, history of referral to psychiatrist, history of consumption depression drugs, percentage of Total Body Surface Area (TBSA) burned history of special disease; and also irrational beliefs and geographical areas had significant relationships with emotional distress. (P< 0.05) Using forward linear regression, gender, irrational beliefs, history of referral to psychiatrist, geographical areas, etiology of burning, environment, duration of burn, history of special disease, body location burned (face only, head and neck, private exposed) and intent of injury were significant predictors of the emotional distress. The models predicted 29 percent (p< 0.001) of emotional distress.

**Conclusions:**

Considering to possible psychiatric morbidity and development of facilities for screening is necessary. Moreover, consultation with mental health experts after burn injuries is highly recommended.

**Keywords:**

Burn, Injuries, GHQ28, Psychological disorders, Disfigurement

